# Supplementary material for: Blocking glycine utilization inhibits multiple myeloma progression by disrupting glutathione balance
Source: Nat Commun. 2022 Jul 11;13:4007. doi: 10.1038/s41467-022-31248-w (PMC9273595; doi:10.1038/s41467-022-31248-w)
Supplement: Supplementary file 1 — Supplementary Information [file 41467_2022_31248_MOESM1_ESM.pdf]

Supplementary Information

Blocking glycine utilization inhibits multiple myeloma progression through disrupting glutathione balance

Jiliang Xia#, Jingyu Zhang#, Xuan Wu, Wanqing Du, Yinghong Zhu, Xing Liu, Zhenhao Liu, Bin Meng, Jiaojiao Guo, Qin Yang, Yihui Wang, Qinglin Wang, Xiangling Feng, Guoxiang Xie, Yi Shen, Yanjuan He, Juanjuan Xiang, Minghua Wu, Gang An, Lugui Qiu, Wei Jia, Wen Zhou*

Inventory of Supplemental Information

Supplemental methods

Supplemental Data for Figures:

Supplementary Fig. 1, related to Figure 1.

Supplementary Fig. 2, related to Figure 2.

Supplementary Fig. 3, related to Figure 3.

Supplementary Fig. 4, related to Figure 4.

Supplementary Fig. 5, related to Figure 5.

Supplementary Fig. 6, related to Figure 6.

Supplementary Fig. 7, related to Figure 7.

Supplemental Tables for Figures:

Supplementary Table S1, related to Figure 1

Supplementary Table S2, related to Methods

Supplementary Table S3, related to Methods

Supplementary Figures

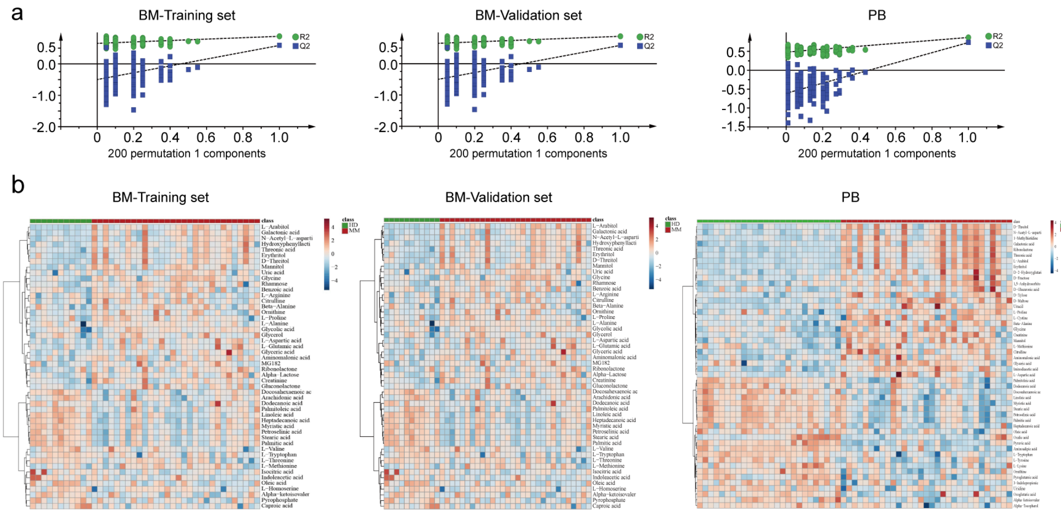

Supplementary Fig. 1 Metabolomic studies in bone marrow liquid and peripheral blood derived from healthy donors and MM patients. a Permutation testing in bone marrow training set, bone marrow validation set, and peripheral blood. **b** Different metabolites identified by univariate statistics (FC > 1.2, $p < 0.05$; unpaired two-sided *t*-test with MetaboAnalyst 5.0 software) in bone marrow training set (left), bone marrow validation set (center), and peripheral blood (right). For samples, green = healthy donors, red = patients with multiple myeloma. For metabolites, blue = decrease; red = increase.

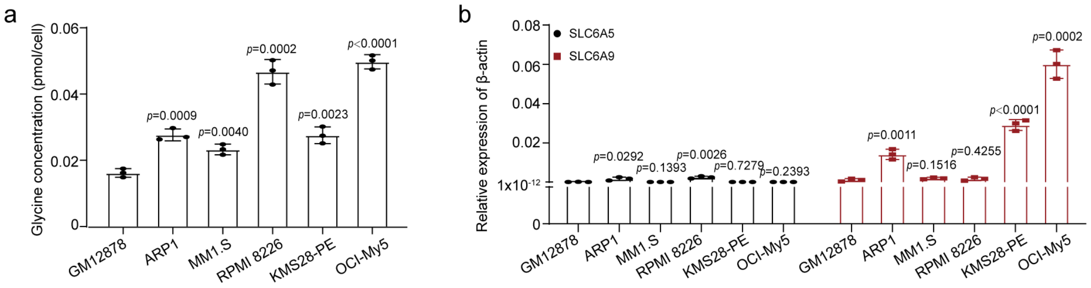

Supplementary Fig. 2 Glycine is increased in MM cell lines compared with GM12878 human B cells. a Glycine concentrations in MM cell lines (ARP1, MM1.S, RPMI 8226, KMS28-PE and OCI-My5) and the human B cell line GM12878. **b** qPCR of *SLC6A5* and *SLC6A9* in MM cell lines and GM12878 human B cells. $n = 3$ independent experiments; Results represent means ± SD; Significance was analyzed with an unpaired two-sided nonparametric *t*-test in **a**, **b.** Source data are provided as a Source Data file.

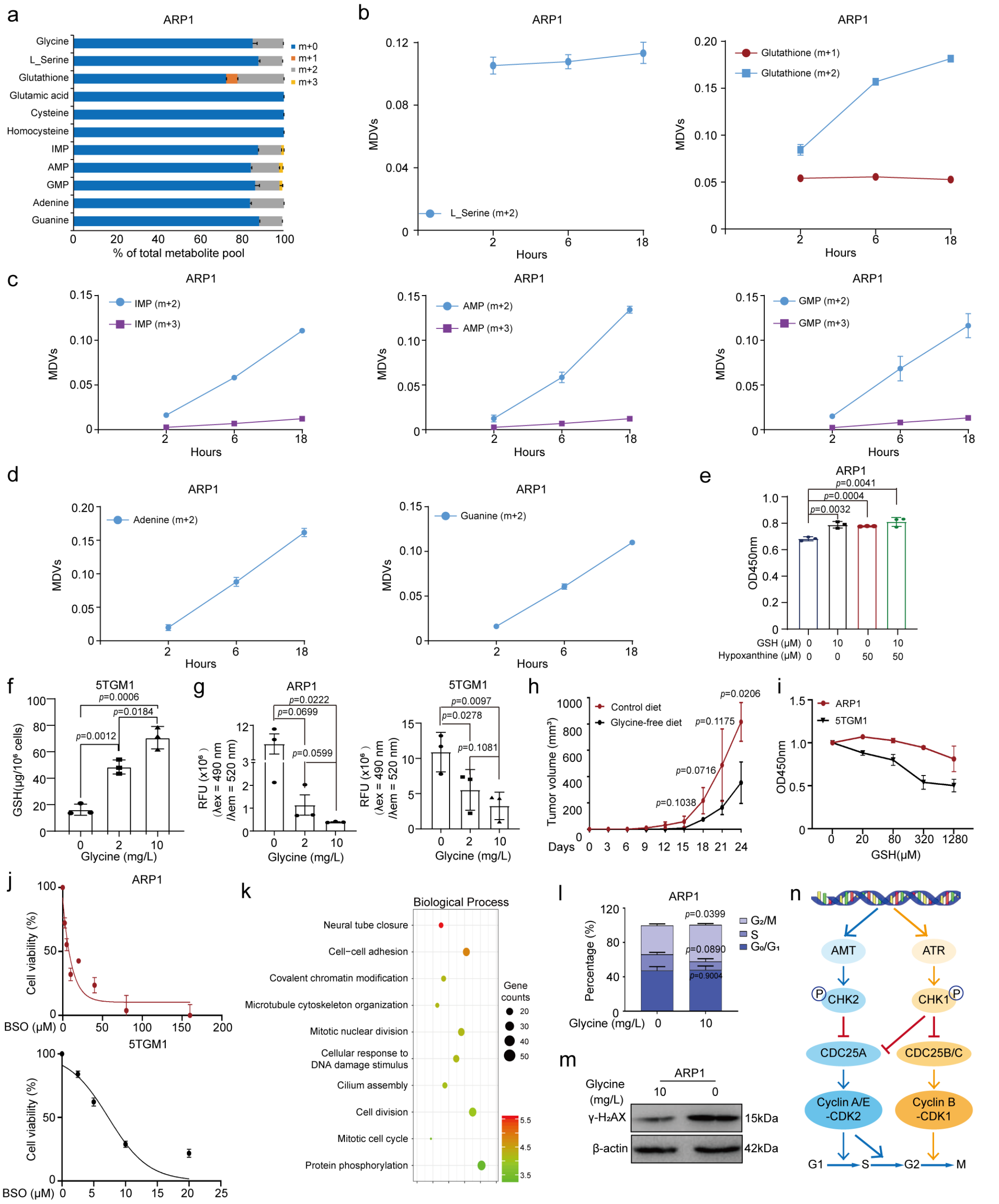

Supplementary Fig. 3 Glycine contributes to proliferation by regulating GSH balance in MM cells. a The fractions of glycine, serine, glutathione, glutamic acid, cysteine, homocysteine, inosine-5′-monophosphate (IMP), adenosine 5-monophosphate (AMP), guanosine 5-monophosphate (GMP), adenine, and guanine containing one (m+1), two (m+2), three (m+3) or zero (m+0) ^{13}C in ARP1 exposed to $^{13}C_2$ glycine for 18 hr. **b** The MDVs of ^{13}C-labeled serine (m+2) and glutathione (m+1, m+2) in ARP1 collected at 2 hr, 6 hr, and 18 hr. **c** The MDVs of IMP (m+2, m+3), AMP (m+2, m+3) and GMP (m+2, m+3) in ARP1 collected at 2 hr, 6 hr, and 18 hr. **d** The MDVs of adenine (m+2) and guanine (m+2) in ARP1 collected at 2 hr, 6 hr, and 18 hr. n = 3 independent experiments; Results represent means ± SD in **a-d**. **e** CCK-8 assays of ARP1 with or without GSH and hypoxanthine. **f** GSH

levels in 5TGM1 cultured with different doses of glycine for 24 hr. **g** ROS levels in ARP1 and 5TGM1 cultured in different doses of glycine for 24 hr. **h** Assessment of tumor volumes in mice fed with glycine-free diet compared with those fed with control diet (n = 3 in each group; Unpaired two-sided nonparametric t-test). **i** CCK-8 assays of ARP1 and 5TGM1 after treatment with GSH (10 μM) for 48 hr. **j** The viability assays of ARP1 and 5TGM1 after treatment with BSO for 48 hr (n = 3 independent experiments; Results represent means ± SD). **k** Analysis of the enrichment of signaling pathways based on differentially expressed genes between ARP1 cultured with or without glycine. **l** The percentages of ARP1 in different cell-cycle phases after culturing with or without glycine for 48 hr. **m** Western blotting of γ-H2AX and β-actin in ARP1 cultured with or without glycine for 48 hr. **n** Schematic of the DNA repair pathway. n = 3 independent experiments; Results represent means ± SD; Unpaired two-sided t-test was used in **e**, **f**, **g**, **i**, **l**. Source data are provided as a Source Data file.

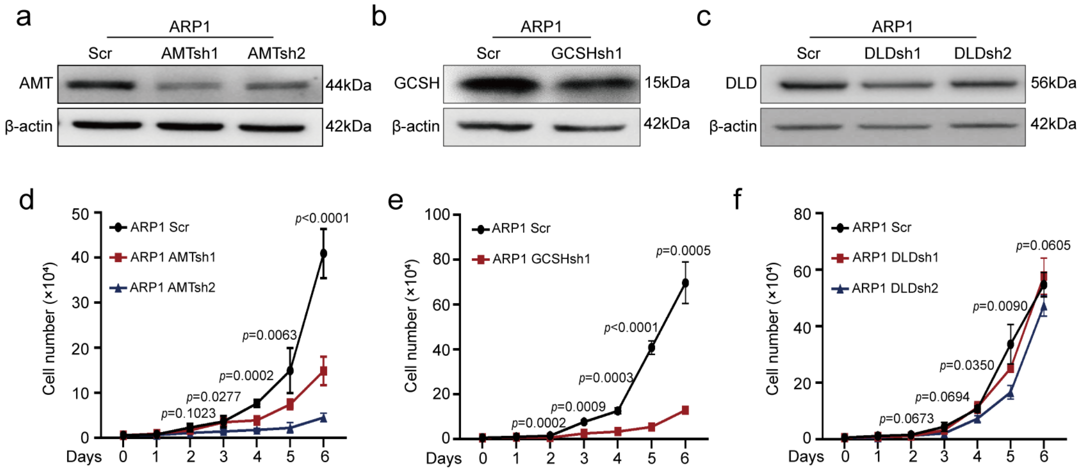

Supplementary Fig. 4 Inhibition of glycine cleavage reduces proliferation in ARP1 cells. a-c Western blotting of AMT, GCSH, DLD, and β-actin in ARP1 AMTsh1, ARP1 AMTsh2, ARP1 GCSHsh1, ARP1 DLDsh1, ARP1 DLDsh2, and ARP1 Scramble (Scr) cells. **d-f** Growth curves of ARP1 AMTsh1, ARP1 AMTsh2, ARP1 GCSHsh1, ARP1 DLDsh1, ARP1 DLDsh2, and ARP1 Scr cells. n = 3 independent experiments; Results represent means ± SD; Unpaired two-sided t-test was applied in **e**; ANOVA one-way test was applied in **d**, **f**. Source data are provided as a Source Data file.

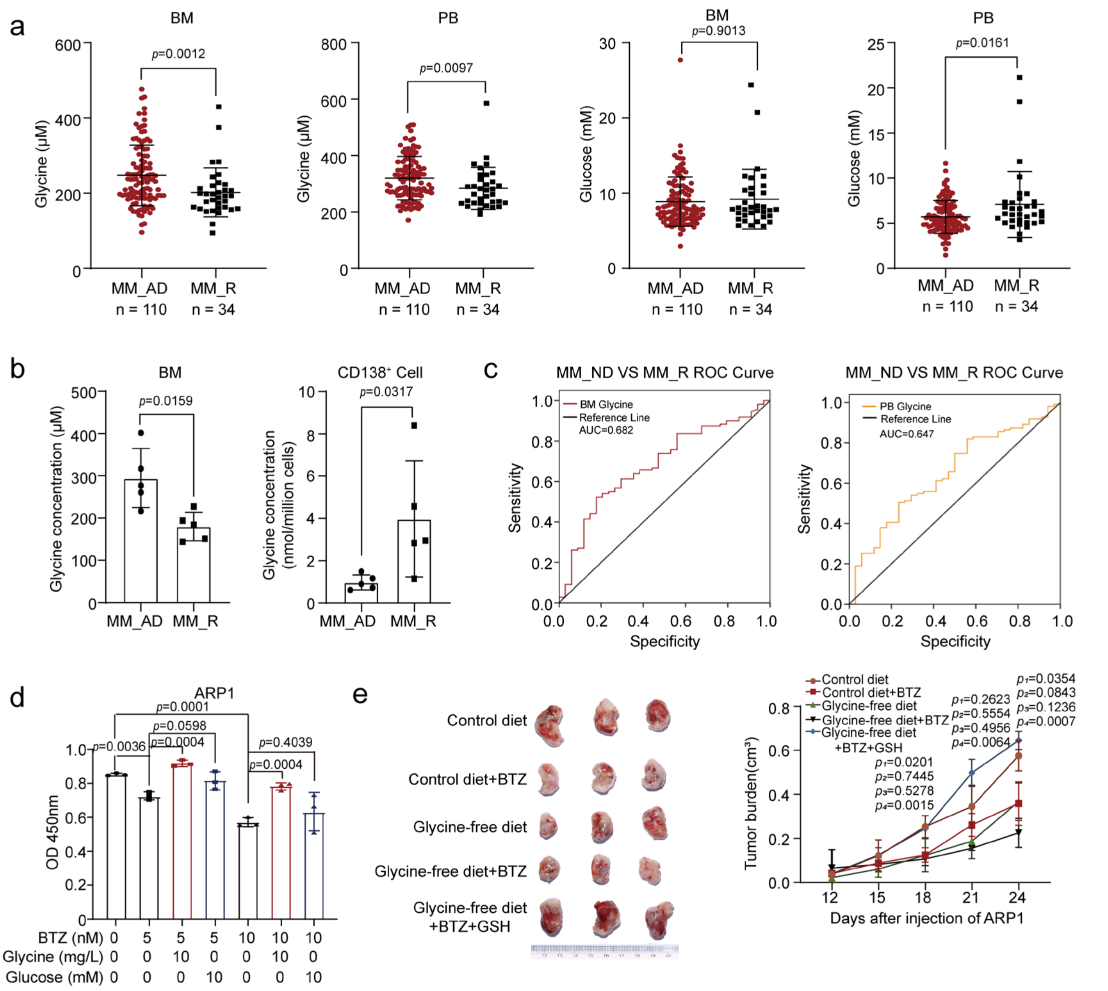

Supplementary Fig. 5 Inhibition of glycine utilization reduces proliferation and enhances the effect of BTZ on MM cells. a The concentrations of glycine and glucose in BM supernatant and PB derived from newly diagnosed MM patients and relapsed MM patients (n = 110 in MM_AD, n = 34 in MM_R; Results represent means ± SD; Unpaired two-sided t-test). **b** Glycine concentrations in the BM supernatant and CD138$^+$ cells of newly diagnosed and relapsed MM patients (n = 5 in MM_AD, n = 5 in MM_R; Results represent means ± SD; Unpaired two-sided t-test). **c** ROC curves drawn based on glycine concentration using subjects derived from newly diagnosed and relapsed MM patients. **d** CCK-8 assay in ARP1 cells treated with glycine (10 mg/L) or glucose (10 mM) for 24 h followed by treatment with BTZ (5 or 10 nM) for 48 hr (n = 3 independent experiments; Results represent means ± SD; Unpaired two-sided t-test). **e** The tumor burden of B-NDG mice fed with control diet (n = 3), fed with control diet and treated with BTZ (1 mg/kg, 3 times/week) (n = 3), fed with glycine-free diet (n = 3), fed with glycine-free diet and treated with BTZ (1 mg/kg, 3 times/week) (n = 3), or fed with glycine-free diet and treated with BTZ (1 mg/kg, 3 times/week) plus GSH (2 mg/kg, 3 times/week) (Results represent means ± SD; Unpaired two-sided t-test, p_1 represent the significance between contro and control + BTZ, p_2 represent the significance between glycine-free and glycine-free + BTZ, p_3 represent the significance between control + BTZ

and glycine-free + BTZ, p_4 represent the significance between glycine-free + BTZ and glycine-free + BTZ + GSH). Source data are provided as a Source Data file.

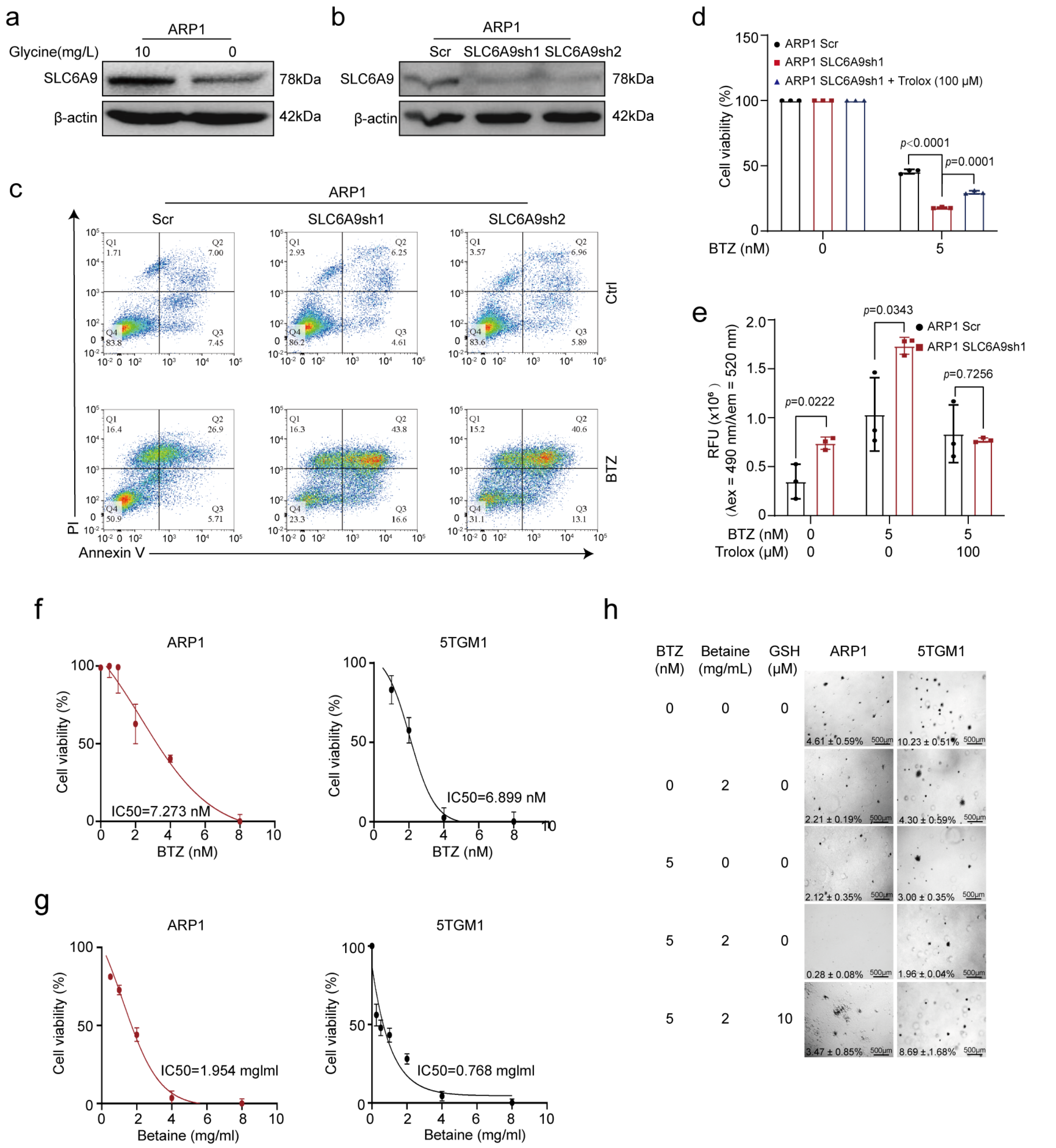

Supplementary Fig. 6 Blocking glycine uptake inhibits cell proliferation and enhances the effects of BTZ on myeloma cells. a Western blotting of SLC6A9 and β-actin in ARP1 cells with or without glycine treatment (10 mg/L). **b** Western blotting of SLC6A9 and β-actin in ARP1 SLC6A9sh1, ARP1 SLC6A9sh2 and ARP1 Scr cells. **c** Analysis of apoptosis via FITC-Annexin V/PI staining in ARP1 Scr, ARP1 SLC6A9sh1, and ARP1 SLC6A9sh2 cells treated with BTZ (5 nM) or up untreated. Cells not stained by PI and Annexin V are non-apoptotic alive cells (Q4). PI positive cells are died cells (Q1, Q2). Cells only stained by Annexin V are at the early stage of apoptosis (Q3). Cells stained by both Annexin V and PI are at the late stage of apoptosis (Q2). **d** CCK-8 assay in ARP1 Scr

and ARP1 SLC6A9sh1 cells exposed to different doses of BTZ (0, 5, 10 nM) and with or without Trolox (100 µM). **e** ROS levels in ARP1 Scr and ARP1 SLC6A9sh1 cells treated with BTZ (5 nM) and with (100 µM) or without Trolox. *n* = 3 independent experiments; Results represent means ± SD; Unpaired two-sided *t*-test was applied in **d**, **e.** **f** The cell viability assays of ARP1 and 5TGM1 cells after treatment with BTZ for 48 hr (*n* = 3 independent experiments; Results represent means ± SD). **g** CCK-8 assays of ARP1 and 5TGM1 cells after treatment with betaine for 48 h (*n* = 3 independent experiments; Results represent means ± SD). **h** Clonogenic analysis of ARP1 or 5TGM1 cells treated with different doses of BTZ (0 or 5 nM) and with or without betaine (2 mg/mL) and glutathione (10 µM) (*n* = 3 independent experiments; Results represent means ± SD). Source data are provided as a Source Data file.

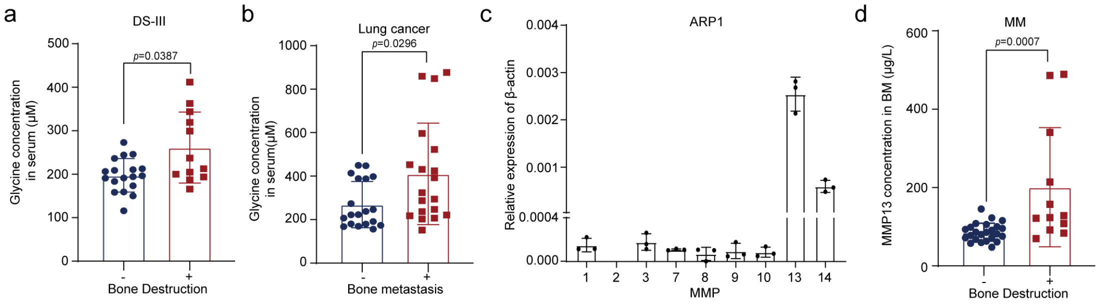

Supplementary Fig. 7 MMP13 is associated with bone destruction in MM. a Serum glycine concentrations in MM patients at DS Stage III with or without bone destruction (*n* = 12 in group with bone destruction, *n* = 18 in group without bone destruction; Results represent means ± SD). **b** Serum glycine concentrations in lung cancer patients with or without bone metastasis (*n* = 19 in group with bone metastasis, *n* = 19 in group without bone metastasis; Results represent means ± SD). **c** qPCR of *MMP1*, *MMP2*, *MMP3*, *MMP7*, *MMP8*, *MMP9*, *MMP10*, *MMP13*, and *MMP14* in ARP1 cells (*n* =3 independent experiments; Results represent means ± SD). **d** The concentrations of MMP13 in BM derived from MM patients with or without bone destruction (*n* = 12 in group with bone destruction, *n* = 24 in group without bone destruction; Results represent means ± SD). Significance was analyzed with an unpaired two-sided *t*-test. Source data are provided as a Source Data file.

Supplementary Tables

Supplementary Table 1 Classes of metabolites detected in multiple myeloma patient samples via untargeted metabolomics assays

Class	BM training set	BM validation set	PB
Amino acid	43	45	41
Carbohydrates	24	25	22
Fatty acids	13	13	14
Organic acids	24	20	21
Lipids	5	5	6
Nucleotide	4	2	3
Alcohols	2	2	2
Aldehydes	1	0	0
Alkylamines	2	1	0
Indoles	1	3	2
Phenols	1	0	1
Phosphate	1	0	0
Vitamin	2	1	1
Total	123	117	113

BM, Bone marrow; PB, Peripheral blood.

Supplementary Table 2 qPCR primer sequences

Target genes	Directions	Sequences
ACTIN	Sense	GTCTTCCCCTCCATCGTG
	Antisense	TTCTCCATGTCGTCCCAG
SLC6A9	Sense	CTGATGCTCCTCACTCTTGGAC
	Antisense	TGCGTAGGTACTTGGGAAACTCG
SLC6A5	Sense	GTCACAGCCATTGTGGATGAGG
	Antisense	GCCGCATAGTTGTCCATCAGCA
MMP1	Sense	ATGAAGCAGCCCAGATGTGGAG
	Antisense	TGGTCCACATCTGCTCTTGGCA
MMP2	Sense	AGCGAGTGGATGCCGCCTTTAA
	Antisense	CATTCCAGGCATCTGCGATGAG
MMP3	Sense	CACTCACAGACCTGACTCGGTT
	Antisense	AAGCAGGATCACAGTTGGCTGG
MMP7	Sense	TCGGAGGAGATGCTCACTTCGA
	Antisense	GGATCAGAGGAATGTCCCATACC
MMP8	Sense	CAACCTACTGGACCAAGCACAC
	Antisense	TGTAGCTGAGGATGCCTTCTCC
MMP9	Sense	GCCACTACTGTGCCTTTGAGTC
	Antisense	CCCTCAGAGAATCGCCAGTACT
MMP10	Sense	TCCAGGCTGTATGAAGGAGAGG
	Antisense	GGTAGGCATGAGCCAAACTGTG
MMP13	Sense	CCTTGATGCCATTACCAGTCTCC
	Antisense	AAACAGCTCCGCATCAACCTGC
MMP14	Sense	CCTTGGACTGTCAGGAATGAGG
	Antisense	TTCTCCGTGTCCATCCACTGGT

Supplementary Table 3 shRNA sequences

Target genes	Directions	Sequences
SLC6A9-1	Sense	CCGGCCCGCCATCATCTTCTTTATTCTCGAGAATAAAGAAGATGATGGCGGGTTTTTG
	Antisense	AATTCAAAAACCCGCCATCATCTTCTTTATTCTCGAGAATAAAGAAGATGATGGCGGG
SLC6A9-2	Sense	CCGGCCGGAACTACTTCCAGGACATCTCGAGATGTCCTGGAAGTAGTTCCGGTTTTTG
	Antisense	AATTCAAAAACCGGAACTACTTCCAGGACATCTCGAGATGTCCTGGAAGTAGTTCCGG
GLDC-1	Sense	CCGGTGTAATCTCTGTCAAGGTAAACTCGAGTTTACCTTGACAGAGATTACATTTTTG
	Antisense	AATTCAAAAATGTAATCTCTGTCAAGGTAAACTCGAGTTTACCTTGACAGAGATTACA
GLDC-2	Sense	CCGGATATTGGCATGGGCTATTATACTCGAGTATAATAGCCCATGCCAATATTTTTTG
	Antisense	AATTCAAAAAATATTGGCATGGGCTATTATACTCGAGTATAATAGCCCATGCCAATAT
DLD-1	Sense	CCGGGCAGTTGAAAGAAGAGGGTATCTCGAGATACCCTCTTCTTTCAACTGCTTTTTG
	Antisense	AATTCAAAAAGCAGTTGAAAGAAGAGGGTATCTCGAGATACCCTCTTCTTTCAACTGC
DLD-2	Sense	CCGGGCAAATCTTGCTGCGTCATTTCTCGAGAAATGACGCAGCAAGATTTGCTTTTTG
	Antisense	AATTCAAAAAGCAAATCTTGCTGCGTCATTTCTCGAGAAATGACGCAGCAAGATTTGC
GCSH	Sense	CCGGCAGGACTTGTAAACAAATCTTCTCGAGAAGATTTGTTTACAAGTCCTGTTTTTG
	Antisense	AATTCAAAAACAGGACTTGTAAACAAATCTTCTCGAGAAGATTTGTTTACAAGTCCTG
AMT-1	Sense	CCGGATATGCTGCAGACCAAGATACCTCGAGGTATCTTGGTCTGCAGCATATTTTTTG
	Antisense	AATTCAAAAAATATGCTGCAGACCAAGATACCTCGAGGTATCTTGGTCTGCAGCATAT
AMT-2	Sense	CCGGTGTGCCCACAAACTACTATACCTCGAGGTATAGTAGTTTGTGGGCACATTTTTG
	Antisense	AATTCAAAAATGTGCCCACAAACTACTATACCTCGAGGTATAGTAGTTTGTGGGCACA
