# Supplementary material for: Blocking glycine utilization inhibits multiple myeloma progression by disrupting glutathione balance
Source: Nat Commun. 2022 Jul 11;13:4007. doi: 10.1038/s41467-022-31248-w (PMC9273595; doi:10.1038/s41467-022-31248-w)
Supplement: Supplementary file 2 — Description of Additional Supplementary Files [file 41467_2022_31248_MOESM2_ESM.pdf]

Description of Additional Supplementary Files

File Name: Supplementary Data 1
Description: Supplementary Data 1 provides the variable importance in projection (VIP), fold change (FC), *p*-value of metabolites obtained from multivariate statistics and univariate statistics based on the abundances of metabolites detected by untargeted metabolomic assays in bone marrow training set, bone marrow validation set peripheral blood set.

File Name: Supplementary Data 2
Description: The list of variable importance in projection (VIP), fold change (FC), *p*-value of different metabolites between healthy donor and patients with multiple myeloma in bone marrow training set, bone marrow validation set, peripheral blood set.

File Name: Supplementary Data 3
Description: Supplementary Data 3 provides the list of different genes between ARP1 cells cultured with or without glycine. The mRNA expression profiling in ARP1 cells cultured with or without glycine for 24 hours was examined by RNA-sequencing. Different genes between ARP1 cells cultured with or without glycine were analyzed by using DESeq2 package in R based on mRNA expression profiling.

File Name: Supplementary Data 4
Description: The list of signaling pathways related to glycine deprivation in ARP1 cells. The Enriched gene ontology (GO) terms were analyzed by using online DAVID software (https://david.ncifcrf.gov/) based on different genes between ARP1 cells cultured with or without glycine.

File Name: Supplementary Data 5
Description: Supplementary Data 5 provides the serum glycine concentrations and clinical characteristics of patients with multiple myeloma in Figure 7 m, d, e, and Supplementary Figure 7a, d. The clinical characteristics include age, gender, DS stage, ISS stage, and bone destruction.
