# Supplementary material for: Blocking glycine utilization inhibits multiple myeloma progression by disrupting glutathione balance
Source: Nat Commun. 2022 Jul 11;13:4007. doi: 10.1038/s41467-022-31248-w (PMC9273595; doi:10.1038/s41467-022-31248-w)
Supplement: Supplementary file 8 — Reporting Summary [file 41467_2022_31248_MOESM8_ESM.pdf]

nature portfolio

Corresponding author(s): Wen Zhou

Last updated by author(s): May 12, 2022

Reporting Summary

Nature Portfolio wishes to improve the reproducibility of the work that we publish. This form provides structure for consistency and transparency in reporting. For further information on Nature Portfolio policies, see our Editorial Policies and the Editorial Policy Checklist.

Statistics

For all statistical analyses, confirm that the following items are present in the figure legend, table legend, main text, or Methods section.

n/a	Confirmed	
☐	☒	The exact sample size (*n*) for each experimental group/condition, given as a discrete number and unit of measurement
☐	☒	A statement on whether measurements were taken from distinct samples or whether the same sample was measured repeatedly
☐	☒	The statistical test(s) used AND whether they are one- or two-sided<br>*Only common tests should be described solely by name; describe more complex techniques in the Methods section.*
☐	☒	A description of all covariates tested
☐	☒	A description of any assumptions or corrections, such as tests of normality and adjustment for multiple comparisons
☐	☒	A full description of the statistical parameters including central tendency (e.g. means) or other basic estimates (e.g. regression coefficient) AND variation (e.g. standard deviation) or associated estimates of uncertainty (e.g. confidence intervals)
☐	☒	For null hypothesis testing, the test statistic (e.g. *F*, *t*, *r*) with confidence intervals, effect sizes, degrees of freedom and *P* value noted<br>*Give P values as exact values whenever suitable.*
☒	☐	For Bayesian analysis, information on the choice of priors and Markov chain Monte Carlo settings
☒	☐	For hierarchical and complex designs, identification of the appropriate level for tests and full reporting of outcomes
☐	☒	Estimates of effect sizes (e.g. Cohen's *d*, Pearson's *r*), indicating how they were calculated

Our web collection on statistics for biologists contains articles on many of the points above.

Software and code

Policy information about availability of computer code

Data collection

1. ChromaTOF software, version 4.71, used for untargeted metabolomics study.
2. SoftMax® Pro 7 software, version 7.1.0 (MolecularDevices), used for ELISA and absorbance.
3. Bruker software, version 4.9 (PerkinElmer), used for whole-animal live imaging.
4. Masslynx software, version 4.1, used for metabolic flux.
5. CFX Manager TM software, version 3.1 (Bio-Rad), used for qRT-PCR.
6. LabSolutions CS software, version 6.90 (Shimadzu), used for HPLC.
7. µCT 40 software, version 1.5 (Scanco Medical), used for Micro-CT.
8. CytExpert software, version 2.0 (Beckman Coulter), used for FACS.
9. SageCapture TM software version 2.17.12.170316 (Sagecreation), used for Western blot.

Data analysis

1. GraphPad Prism version 7 and SPSS version 20.0 was used for data analysis.
2. SIMCA 14.1 software package was used for orthogonal partial least-squares-discriminant analysis (OPLS-DA) in untargeted metabolomics study.
3. IBM SPSS Statistics software, version 20.0 was used for operator characteristic curve (ROC) analyses.
4. Metabolites in untargeted metabolomics study were annotated with the mammalian metabolite database JiaLibTM using a strict-matching algorithm incorporated in XploreMET software, version 3.0.
5. Metabolites in untargeted metabolomics study were analyzed at the univariate level by using MetaboAnalyst 5.0.
6. The data of metabolic flux were analyzed by using Targetlynx software, version 4.1 software.
7. ImageJ software, version 1.8.0 was used for IF.
8. µCT data was analyzed by CTAn version 1.11 (Bruker) and µCTVol version 2.2 (Bruker).
9. The data of flow cytometry was analyzed by FlowJo, version 10.0.7 (BD)

For manuscripts utilizing custom algorithms or software that are central to the research but not yet described in published literature, software must be made available to editors and reviewers. We strongly encourage code deposition in a community repository (e.g. GitHub). See the Nature Portfolio guidelines for submitting code & software for further information.

Data

Policy information about availability of data

All manuscripts must include a data availability statement. This statement should provide the following information, where applicable:

- Accession codes, unique identifiers, or web links for publicly available datasets
- A description of any restrictions on data availability
- For clinical datasets or third party data, please ensure that the statement adheres to our policy

The raw data of RNA-sequencing reported in this study has been deposited in the public database of Genome Sequence Archive (GSA) in National Genomics Data Center under the accession number HRA002453, which is accessible at https://ngdc.cncb.ac.cn/gsa-human/. The untargeted metabolomics data was included in an Excel form which is named as untargeted metabolomic data in the Source Data file. Source data are provided in Source Data file.

Field-specific reporting

Please select the one below that is the best fit for your research. If you are not sure, read the appropriate sections before making your selection.

☒ Life sciences ☐ Behavioural & social sciences ☐ Ecological, evolutionary & environmental sciences

For a reference copy of the document with all sections, see nature.com/documents/nr-reporting-summary-flat.pdf

Life sciences study design

All studies must disclose on these points even when the disclosure is negative.

Sample size	No Sample size calculation was performed in this study. Sample size was determined according to previous publications. Reference: Jian X, et al. Alterations of gut microbiome accelerate multiple myeloma progression by increasing the relative abundances of nitrogen-recycling bacteria. Microbiome. 8(1):74 (2020).
Data exclusions	No data were excluded from the analyses.
Replication	All data presented were from biological replicates. Data described in this manuscript were reliably reproduced.
Randomization	Samples and mice were randomly allocated to control or experimental groups. Multiple myeloma patients, lung cancer patients and healthy donors were randomly selected in this study. These multiple myeloma patients were divided into two groups (27 patients in low glycine group, 83 patients in high glycine group) based on the concentration of glycine in bone marrow supernatant (Table 1). Multiple myeloma patients in Fig 7c were divided into two groups (24 patients with bone destruction, 12 patients without bone destruction) based on bone destruction. Multiple myeloma patients at DS-III stage in Extended Data Fig 7a were divided into two groups (12 patients with bone destruction, 18 patients without bone destruction) based on bone destruction. Lung cancer patients in Extended Data Fig 7b were divided into two groups (19 patients with bone metastasis, 19 patients without bone metastasis) based on bone metastasis. Multiple myeloma patients in Extended Data Fig 7d were divided into two groups (12 patients with bone destruction, 24 patients without bone destruction) based on bone destruction.
Blinding	Investigators were not blinded to sample allocation during experiment and outcome assessment. However, the experimenter were blinded during data collection and analyses.

Reporting for specific materials, systems and methods

We require information from authors about some types of materials, experimental systems and methods used in many studies. Here, indicate whether each material, system or method listed is relevant to your study. If you are not sure if a list item applies to your research, read the appropriate section before selecting a response.

Materials & experimental systems

n/a	Involved in the study	
☐	☒	Antibodies
☐	☒	Eukaryotic cell lines
☒	☐	Palaeontology and archaeology
☐	☒	Animals and other organisms
☐	☒	Human research participants
☒	☐	Clinical data
☒	☐	Dual use research of concern

Methods

n/a	Involved in the study	
☒	☐	ChIP-seq
☐	☒	Flow cytometry
☒	☐	MRI-based neuroimaging

Antibodies

Antibodies used

1. SLC6A9 Rabbit Polyclonal Antibody, Cat#A16203 , Western Blotting 1:1000, ABclonal.
2. GLDC Rabbit Polyclonal Antibody, Cat#24827-1-AP, Western Blotting 1:1000, Proteintech Group.
3. GCSH Rabbit Polyclonal Antibody, Cat#16726-1-AP, Western Blotting 1:1000, Proteintech Group.
4. DLD Rabbit Polyclonal Antibody, Cat#16431-1-AP, Western Blotting 1:1000, Proteintech Group.
5. AMT Rabbit Polyclonal Antibody, Cat#A9926, Western Blotting 1:1000, ABclonal.
6. CDC25A Rabbit Polyclonal Antibody, Cat#55031-1-AP, Western Blotting 1:1000, Proteintech Group.
7. Beta Actin Mouse Monoconal antibody, Cat#66009-1-Ig, Clone 2D4H5, Western Blotting 1:5000, Proteintech Group.
8. Phospho-ATR (Ser428) Rabbit Polyclonal Antibody, Cat#2853S, Western Blotting 1:1000, Cell Signaling Technology.
9. Phospho-ATM (Ser1981) Rabbit Monoconal Antibody, Cat#5883S, Clone D6H9, Western Blotting 1:1000, Cell Signaling Technology.
10. Phospho-Chk1 (Ser345) Rabbit Monoclonal Antibody, Cat#2348S, Clone 133D3, Western Blotting 1:1000, Cell Signaling Technology.
11. Phospho-Chk2 (Thr68) Rabbit Polyclonal Antibody, Cat#2197S, Western Blotting 1:1000, Cell Signaling Technology.
12. Phospho-Histone H2A.X (Ser139) Rabbit Polyclonal Antibody, Cat#9718S, Immunofluorescence 1:100, Western Blotting 1:1000, Cell Signaling Technology.
13. Cleaved Capase-3 (Asp175) Rabbit Polyclonal Antibody, Cat#9661S, Western Blotting 1:1000, Cell Signaling Technology.
14. PARP Rabbit Polyclonal Antibody, Cat#9542S, Western Blotting 1:1000, Cell Signaling Technology.
15. HRP-conjugated goat anti-mouse IgG secondary antibody, Cat#101, Western Blotting 1:5000, Signalway Antibody.
16. HRP-conjugated goat anti-rabbit IgG secondary antibody, Cat#L3042, Western Blotting 1:5000, Signalway Antibody.

Validation

All primary antibodies are commercially available and were validated by the manufacturer as follows:
1. SLC6A9 Rabbit Polyclonal Antibody, Cat#A16203, ABclonal. The antibody was validated by Western Blotting in 4 cell lines and 2 tissues, including SH-SY5Y, K-562, MCF7, C6, mouse liver, and rat live. The figure of Western Blotting is listed on the manufacturer's website. (https://abclonal.com.cn/catalog/A16203).
2. GLDC Rabbit Polyclonal Antibody, Cat#24827-1-AP, Proteintech Group. The manufacturer states that this antibody can be used for Western Blotting, IP, IHC, ELISA. The antibody was used in 2 publications (https://www.ptglab.com/products/GLDC-Antibody-24827-1-AP.htm).
3. GCSH Rabbit Polyclonal Antibody, Cat#16726-1-AP, Proteintech Group. The manufacturer states that this antibody can be used for Western Blotting, IP, IHC, IF, ELISA. The antibody was used in 7 publications (https://www.ptglab.com/products/GCSH-Antibody-16726-1-AP.htm).
4. DLD Rabbit Polyclonal Antibody, Cat#16431-1-AP, Proteintech Group. The manufacturer states that this antibody can be used for Western Blotting, IP, IHC, IF, ELISA. The antibody was used in 5 publications (https://www.ptglab.com/products/DLD-Antibody-16431-1-AP.htm).
5. AMT Rabbit Polyclonal Antibody, Cat#A9926, ABclonal. The antibody was validated by Western Blotting, IHC and IF. The figure of Western Blotting is listed on the manufacturer's website (https://abclonal.com.cn/catalog/A9926).
6. CDC25A Rabbit Polyclonal Antibody, Cat#55031-1-AP, Proteintech Group. The manufacturer states that this antibody can be used for Western Blotting, IP, IHC, IF, ELISA. The antibody was used in 20 publications (https://www.ptglab.com/products/CDC25A-Antibody-55031-1-AP.htm).
7. Beta Actin Mouse Monoconal antibody, Cat#66009-1-Ig, Proteintech Group. The manufacturer states that this antibody can be used for Western Blotting, IP, IHC, IF, FC, CoIP, ChIP, ELISA. The antibody was used in 2789 publications (https://www.ptglab.com/products/Pan-Actin-Antibody-66009-1-Ig.htm).
8. Phospho-ATR (Ser428) Rabbit Polyclonal Antibody, Cat#2853S, Cell Signaling Technology. The manufacturer states that this antibody can be used for Western Blotting. The antibody was used in 211 publications (https://www.cellsignal.com/products/primary-antibodies/phospho-atr-ser428-antibody/2853?site-search-type=Products&N=4294956287&Ntt=phospho-atr+%28ser428%29+&fromPage=plp&_requestid=1329919).
9. Phospho-ATM (Ser1981) Rabbit Monoconal Antibody, Cat#5883S, Clone D6H9, Cell Signaling Technology. The manufacturer states that this antibody can be used for Western Blotting. The antibody was used in 166 publications (https://www.cellsignal.com/products/primary-antibodies/phospho-atm-ser1981-d6h9-rabbit-mab/5883?site-search-type=Products&N=4294956287&Ntt=phospho-atm+%28ser1981%29&fromPage=plp).
10. Phospho-Chk1 (Ser345) Rabbit Polyclonal Antibody, Cat#2348S, Cell Signaling Technology. The manufacturer states that this antibody can be used for Western Blotting. The antibody was used in 323 publications (https://www.cellsignal.com/products/primary-antibodies/phospho-chk1-ser345-antibody/2341?site-search-type=Products&N=4294956287&Ntt=phospho-chk1+%28ser345%29&fromPage=plp).
11. Phospho-Chk2 (Thr68) Rabbit Polyclonal Antibody, Cat#2197S, Cell Signaling Technology. The manufacturer states that this antibody can be used for Western Blotting, IF, FC. The antibody was used in 534 publications (https://www.cellsignal.com/products/primary-antibodies/phospho-chk2-thr68-antibody/2661?site-search-type=Products&N=4294956287&Ntt=phospho-chk2+%28thr68%29+&fromPage=plp).
12. Phospho-Histone H2A.X (Ser139) Rabbit Polyclonal Antibody, Cat#9718S, Cell Signaling Technology. The manufacturer states that this antibody can be used for Western Blotting, IF, FC. The antibody was used in 751 publications (https://www.cellsignal.com/products/primary-antibodies/phospho-histone-h2a-x-ser139-antibody/2577?site-search-type=Products&N=4294956287&Ntt=phospho-histone+h2a.x+%28ser139%29&fromPage=plp).
13. Cleaved Capase-3 (Asp175) Rabbit Polyclonal Antibody, Cat#9661S, Cell Signaling Technology. The manufacturer states that this antibody can be used for Western Blotting, IF, IP, IHC, FC. The antibody was used in 7055 publications (https://www.cellsignal.com/products/primary-antibodies/cleaved-caspase-3-asp175-antibody/9661?site-search-type=Products&N=4294956287&Ntt=cleaved+capase-3+%28asp175%29+&fromPage=plp).
14. PARP Rabbit Polyclonal Antibody, Cat#9542S, Cell Signaling Technology. The manufacturer states that this antibody can be used for Western Blotting. The antibody was used in 3010 publications (https://www.cellsignal.com/products/primary-antibodies/parp-antibody/9542?site-search-type=Products&N=4294956287&Ntt=parp+rabbit+polyclonal+antibody%2C+cat%239542s&Nrpp=30&No=30&fromPage=plp).
15. HRP-conjugated goat anti-mouse IgG secondary antibody, Cat#101, Signalway Antibody. The manufacturer states that this antibody can be used for WB, ELISA (https://www.sabbiotech.com.cn/g-229544-HRP-conjugated-goat-anti-mouse-IgG-secondary-antibody-101.html) .

16. HRP-conjugated goat anti-rabbit IgG secondary antibody, Cat#L3042, ignalway Antibody. The manufacturer states that this antibody can be used for WB, IHC, ELISA. The antibody was used in 1 publication (https://www.sabbiotech.com.cn/g-3812-Rabbit-anti-Goat-IgG-Secondary-Antibody-HRP-conjugated-L3042.html).

Eukaryotic cell lines

Policy information about cell lines

Cell line source(s)	Cell lines including ARP1, 5TGM1, MM1.S, KMS28-PE, OCI-My5, RPMI 8226, GM12878, HEK293T were obtained from Cancer Research Institute, Central South University.
Authentication	All cell lines were authenticated by STR DNA profiling analysis.
Mycoplasma contamination	Cell lines used in this study are negative for mycoplasma.
Commonly misidentified lines (See ICLAC register)	No commonly misidentified cell lines used in this study.

Animals and other organisms

Policy information about studies involving animals; ARRIVE guidelines recommended for reporting animal research

Laboratory animals	6-8-week-old male or female C57BL/KaLwRij mice and B-NDG (NOD.CB17-Prkdcscidll2rgtm1/Bcgen) mice were used in this study. All mice were maintained under SPF conditions in a controlled environment of 20–22 °C, with a 12/12 h light/dark cycle, 50–70% humidity.
Wild animals	No wild animals used in this study.
Field-collected samples	No field-collected samples were used.
Ethics oversight	All animal experiments were performed in accordance with the guidelines of the Institutional Animal Care and Local Veterinary Office and Ethics Committee of the CSU and the Hunan Normal University, China (animal experimental license number for B-NDG mice: NO.2019sydw0154 and for C57BL/KalwRijHsd mice: D2020001).

Note that full information on the approval of the study protocol must also be provided in the manuscript.

Human research participants

Policy information about studies involving human research participants

Population characteristics	All participants are from China. The median age of health doners is 67 (range 25-88) and 26.67% of these patients are male. The median age of multiple myeloma patients is 58 (range 34-81) and 51.82% of these patients are male. The median age of lung cancer patients is 66 (range 52-81) and 78.95% of these patients are male.
Recruitment	The patients and healthy donors were randomly recruited at Xiangya Hospital, the Second, the Third Xiangya Hospital of Central South University and the Blood Diseases Hospital of Chinese Academy of Medical Science & Peking Union Medical College. There is no potential self-selection bias or other biases.
Ethics oversight	Ethics approval was granted by the Cancer Research Institute Review Board of Central South University.

Note that full information on the approval of the study protocol must also be provided in the manuscript.

Flow Cytometry

Plots

Confirm that:

☒ The axis labels state the marker and fluorochrome used (e.g. CD4-FITC).

☒ The axis scales are clearly visible. Include numbers along axes only for bottom left plot of group (a 'group' is an analysis of identical markers).

☒ All plots are contour plots with outliers or pseudocolor plots.

☒ A numerical value for number of cells or percentage (with statistics) is provided.

Methodology

Sample preparation	The apoptosis assay was descriptived in "Supplemental methods" section
Instrument	DxP AthenaTM Flow Cytometer (Cytek)

Software	FlowJo, version 10.0.7 (BD)
Cell population abundance	This study is not involved with Cell sorting procedure.
Gating strategy	To identify the cells population of interesting, the forward vs side scatter (FSC-A versus SSC-A) was used. The FSC-A/FSC-H were used to remove the doublets from total cell population. Apoptotic cells were gated out based on gating of unstained cells and single stained cells using appropriate channels.

☒ Tick this box to confirm that a figure exemplifying the gating strategy is provided in the Supplementary Information.
